# Une rare cause de sciatique non discale: une polyarthrite rhumatoïde

**DOI:** 10.11604/pamj.2014.19.76.3621

**Published:** 2014-09-24

**Authors:** Nessrine Akasbi, Fatima Ezzahra Abourazzak, Taoufik Harzy

**Affiliations:** 1 Service de Rhumatologie, CHU Hassan II Fès, Maroc

**Keywords:** Sciatique non discale, polyarthrite rhumatoïde, neuropathies, Non discal sciatica, rheumatoid arthritis, neuropathies

## Abstract

**La polyarthrite rhumatoïde** est le rhumatisme inflammatoire chronique le plus fréquent. Ses complications neurologiques sont dominées par les neuropathies périphériques, cliniquement asymptomatiques, mais peuvent être détectées sur l’électromyogramme (EMG). Nous rapportons l'observation d'une patiente qui a présenté une neuropathie sensitivo-motrice aux deux membres inférieurs associée à une sciatique de topographie L5 d'origine rhumatoïde.

## Aux editeurs du Journal Panafricain de Médecine

Nous rapportons le cas d'une patiente âgée de 59 ans, suivie **pour une** polyarthrite rhumatoïde (PR) séronégative pour le **facteur rhumatoïde**, à anticorps anti-cyclic citrullinated peptide (anti CCP) positif, évoluant depuis 10 ans, évolutive et érosive mise sous traitement de fond à type de méthotrexate 15 mg/semaine depuis 7 ans avec une bonne réponse thérapeutique. La patiente a présenté depuis 2 ans une sciatique L5 droite permanente. L'examen clinique n'a pas trouvé de déficit sensitivo-moteur à l'examen neurologique avec un testing musculaire normale. Devant la résistance au traitement classique et l'aggravation de l'intensité de la douleur, une imagerie par résonnance magnétique médullaire et du pelvis a été réalisé, celle-ci n'a pas objectivé un conflit disco radiculaire ou de cause de **compression du nerf** sciatique. Un électromyogramme (EMG) a montré une atteinte radiculaire L5 droite associée à une atteinte axonale sensitivo-motrice bilatérale, symétrique et diffuse aux deux membres inférieurs. Dans les antécédents de la patiente, on ne note pas de chirurgie pelvienne, ni de traumatisme, pas d'alcoolisme, et pas de maladie métabolique telle que le diabète. Un bilan a été réalisé pour écarter une origine toxicocarentielle, métabolique, infectieuse et néoplasique qui pourrait expliquer cette neuropathie, celui-ci est revenu normale. Donc, on a rattaché la sciatique et la polyneuropathie diffuse à sa polyarthrite rhumatoïde. La patiente a été mise sous faible dose de prédnisone 10 mg/jour associé à l'amitriptyline 10 gouttes le soir avec un bon contrôle de douleur. La PR est le rhumatisme inflammatoire chronique le plus fréquent. Les complications neurologiques de la PR sont les moins fréquentes des manifestations extra articulaires. Elles sont dominées par les neuropathies périphériques, cliniquement asymptomatiques, mais peuvent être détectées sur l'EMG, la compression médullaire par la luxation atloidoaxoidienne et la vascularite rhumatoïde cérébrale. La neuropathie rhumatoïde est dominée par les syndromes canalaires, les polyneuropathies et les mononeuropathies multiples [1]. A notre connaissance, il s'agit du premier cas de sciatique non discale comme manifestation extra articulaire de la PR. Dans notre cas la souffrance du nerf grand sciatique traduit très probablement l'inflammation **chronique** due aux cytokines pro inflammatoires qui génèrent un œdème neuronale, à la vascularite rhumatoïde avec une fibrose et une nécrose périvasculaire et une prolifération intimale provoquant une ischémie du nerf, ou encore une infiltration amyloïde dans l'endoneurium et l’épineuriume du **nerf sciatique**. L'atteinte neurologique peut être aussi expliqué dans certains cas par la présence de synovite articulaire, ou de ténosynovite à l'origine de la compression neurologique et des nodules rhumatoïdes, [2] ou de neurotoxicité des traitements immunosuppresseurs tel que les anti-TNFa ou le léflunomide [3, 4]. Selon les études, il n'y a pas de corrélation entre l’**atteinte** neurologique au cours de la PR et la durée d’évolution, l'activité de la PR, le titre d'anti CCP et la présence d’érosion osseuse [5]. La prise en charge de la **neuropathie** rhumatoïde se base sur un traitement symptomatique associé à un traitement **de fond** de la polyarthrite rhumatoïde.
